# Poultry Food Assess Risk Model for *Salmonella* and Chicken Eggs in Riyadh, Saudi Arabia

**DOI:** 10.3390/foods14193382

**Published:** 2025-09-30

**Authors:** Amani T. Alsufyani, Norah M. Alotaibi, Fahad M. Alreshoodi, Lenah E. Mukhtar, Afnan Althubaiti, Manal Almusa, Maha Althubyani, Rashed Bin Jaddua, Bassam Alsulaiman, Sarah Alsaleh, Saleh I. Alakeel, Thomas P. Oscar, Sulaiman M. Alajel

**Affiliations:** 1 Saudi Food and Drug Authority (SFDA), Riyadh 13513, Saudi Arabia; atsufyani@sfda.gov.sa (A.T.A.); noramajd22@gmail.com (N.M.A.); fmreshoodi@sfda.gov.sa (F.M.A.); afnan.althob@gmail.com (A.A.); mamusa@sfda.gov.sa (M.A.); maha-althubyani@hotmail.com (M.A.); rashed.a.jaddua@gmail.com (R.B.J.); bassamalsuliman@gmail.com (B.A.); alsalehasarah@gmail.com (S.A.); siakeel@sfda.gov.sa (S.I.A.); sulaimanalajel@gmail.com (S.M.A.); 2 United States Department of Agriculture, Agricultural Research Service, Northeast Region, Eastern Regional Research Center, Microbial and Chemical Food Safety Research Unit, University of Maryland Eastern Shore Worksite, Princess Anne, MD 21853, USA; tptoscar@aol.com

**Keywords:** salmonellosis, quantitative microbial risk assessment, consumer survey, prevalence, number, serotype

## Abstract

*Salmonella* presents serious risks to human health, causing about 150,000 deaths per year through the consumption of contaminated food, especially chicken eggs. Consequently, risk of salmonellosis from chicken eggs is of significant interest to the Saudi Food and Drug Authority (SFDA). Models that predict the risk of salmonellosis from chicken eggs are valuable tools for protecting public health. After a review of existing models, the SFDA selected the Poultry Food Assess Risk Model (PFARM) for the purpose of evaluating its ability to assess the risk and severity of salmonellosis for a small cohort of chicken egg consumers in Riyadh, Saudi Arabia, as a proof-of-concept and pilot study. The PFARM was selected because it uses novel methods to consider more risk factors for salmonellosis than other models, such as growth potential and zoonotic potential of *Salmonella*, buffering capacity of the meal, and consumer behavior, health, and immunity. The SFDA examined chicken eggs from retail stores in Riyadh for *Salmonella* contamination and surveyed 125 consumers to obtain data for simulating how they store, prepare, and consume eggs at home, and their resistance to salmonellosis. The prevalence of *Salmonella* in chicken eggs at retail was 7% (7/100). The isolated *Salmonella* serotypes were Cerro (*n* = 4), Enteritidis, Stanley, and Winston. *Salmonella*’s mean number (growth units) per contaminated egg was 1.58 log_10_ (range: 0 to 3.08 log_10_). The mean category for consumer survey results ranged from 1.1 (very low risk) for meal preparation time to 3.7 (high risk) for home storage time with 34.4% of consumers having low resistance to salmonellosis. Per 100,000 egg meals, the PFARM predicted 88 infections, two illnesses, and no hospitalizations or deaths. The consumers who became ill were exposed to *Salmonella* Enteritidis, had moderate resistance to salmonellosis but high-risk behaviors for egg storage (temperature abuse), meal preparation (poor hygiene), and consumption (undercooked eggs). These results showed that the studied chicken eggs posed a low risk and severity of salmonellosis for the surveyed consumer cohort in Riyadh, Saudi Arabia, and that the PFARM was fit-for-purpose. The next step is to improve the PFARM and apply it more broadly in Saudi Arabia to better define the problem and its control.

## 1. Introduction

*Salmonella* is recognized as a worldwide human health concern causing about 150,000 deaths per year through the consumption of contaminated food, especially chicken eggs [1,2]. However, in the Gulf Cooperation Council countries (Bahrain, Kuwait, Oman, Qatar, Saudi Arabia, and United Arab Emirates), less is known about the economic and human health impacts of salmonellosis and its attribution to specific food sources like chicken eggs [3]. Consequently, food safety standards for chicken eggs do not exist in all countries, like Saudi Arabia.

In Saudi Arabia, chicken eggs are a fundamental part of the diet [4]. This is because of their high nutritional value, versatility in cooking, and availability through retail markets [3]. Hence, the potential risk of salmonellosis from eating chicken eggs has attracted significant interest from the Saudi Food and Drug Authority (SFDA). Moreover, antibiotic resistance, which annually kills nearly five million worldwide, is observed in human clinical cases of salmonellosis from chicken eggs in Saudi hospitals making it imperative to properly monitor its occurrence [5]; especially considering the World Health Organization inclusion of fluoroquinolone-resistant non-typhoidal *Salmonella* in the high risk group of the Bacterial Pathogen Priority List [6]. Each of these factors increases the likelihood of a widespread outbreak of salmonellosis with an increased severity due to antibiotic therapy failure [7]. Thus, it is essential to investigate the risk and severity of salmonellosis from chicken eggs in Saudi Arabia.

Models that predict the risk of salmonellosis from chicken eggs are valuable tools for assessing this risk to human health [8,9,10]. However, current models for *Salmonella* and chicken eggs are based on data collection and modeling methods that do not accurately predict the risk of salmonellosis, leading to the wrong food safety decision, as shown in a recent study [11]. Notably, they do not consider important risk factors for salmonellosis like the growth potential and zoonotic potential of *Salmonella* serotypes, buffering ability of the meal, consumer health and immunity, and severity of salmonellosis. Also, the modeling methods used in existing models [8,9,10] overestimate exposure to *Salmonella* for the 13 reasons stated in [12] and risk of salmonellosis for the reasons stated in [11], requiring the use of correction factors to make predictions agree with epidemiologic data [13]. However, new and more accurate data collection and modeling methods are used in the Poultry Food Assess Risk Model (PFARM) for predicting the risk and severity of salmonellosis from poultry food without using correction factors as reviewed in recent studies [12,14,15,16]. These include, but are not limited to, whole sample enrichment, quantitative real-time polymerase chain reaction for *Salmonella* enumeration and prediction of dose–response as a function of zoonotic potential of *Salmonella* serotypes, buffering capacity of the meal, and consumer health and immunity.

The PFARM has only be used in the United States and for assessing the risk of salmonellosis from poultry meat [11,16,17,18] and not chicken eggs. Thus, in the present study, the PFARM was selected by SFDA over other existing models to evaluate its ability to assess the risk and severity of salmonellosis for a small group of chicken egg consumer in Riyadh, Saudi Arabia, as a proof-of-concept and pilot study. This involved examining eggs for *Salmonella* at retail using the PFARM methods, as well as using the PFARM survey to gather information about consumer health and immunity, and how consumers store, prepare, and eat chicken eggs at home.

This was a proof-of-concept and fit-for-purpose study and not a study aimed at setting a national food safety standard in Saudi Arabia or to compare results to other cities or countries, which would require national, regional, and international data, respectively, rather than the local data collection plan used in this study. In other words, the current study was limited in geographic scope and used a relatively small cohort of consumers and egg samples. Therefore, it was not generalizable to the whole population of Saudi Arabia or to the population of another city or nation. Rather, it serves as a pilot study for a future regional, nationwide, or international study. Consequently, it was designed to be of sufficient scope to address the objective of the study, which was to demonstrate that the data collection and modeling methods of the PFARM for chicken meat and *Salmonella* could be used to assess the risk and severity of salmonellosis from chicken eggs in Riyadh, Saudi Arabia. Of note, this is the first study to use data from the consumer survey in the PFARM to assess the risk and severity of salmonellosis from poultry food. It is also the first study outside of the United States to use the PFARM data collection and modeling methods to assess the risk and severity of salmonellosis from a poultry food. Thus, successful attainment of the study objective was important because it demonstrated that the PFARM has broader application than the United States and poultry meat.

## 2. Materials and Methods

**Figure 1:**
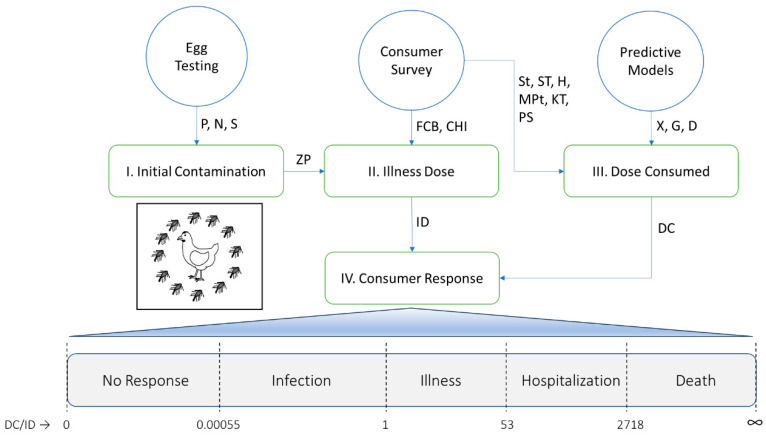
Flow diagram of the Poultry Food Assess Risk Model (PFARM) for *Salmonella* and chicken eggs in Riyadh, Saudi Arabia, showing how the data collection (egg testing and consumer survey) and predictive modeling steps flow into the four steps (initial contamination, illness dose, dose consumed, and consumer response) of the PFARM to predict health outcomes (no response, infection, illness, hospitalization, and death) as a function of the ratio of dose consumed to illness dose (DC/ID); see text for details. Abbreviations: P = prevalence; N = number; S = serotype; ZP = zoonotic potential; FCB = food consumption behavior; CHI = consumer health and immunity; St = storage time; ST = storage temperature; H = hygiene; MPt = meal preparation time; KT = kitchen temperature; PS = portion size; X = cross-contamination; G = growth; D = death; ID = illness dose; and DC = dose consumed.

Figure 1 shows how the data collection and predictive modeling steps in the Poultry Food Assess Risk Model (PFARM) for *Salmonella* and chicken eggs in Riyadh, Saudi Arabia, flow into the four steps of the PFARM process: initial contamination [14], illness dose [15], dose consumed [12], and consumer response [16]. The PFARM with its coding is provided as a Supplemental File. Detailed flow diagrams of the four steps of the PFARM are provided in recent studies [12,14,15,16] for better understanding of the methods.

### 2.1. Data Collection Methods

The SFDA purchased 50 plates of 30 chicken eggs from retail markets in Riyadh, Saudi Arabia, on four dates in 2023: April 4th (5 plates), May 8th (15 plates), May 15th (13 plates), and May 22nd (17 plates). The eggs were representative of the brands purchased by the consumer cohort studied in the Riyadh retail market. The SFDA transported eggs at air temperature to the reference laboratory for microbiology at the SFDA. Upon arrival, the SFDA stored the eggs at 4 to 6 °C prior to analysis for *Salmonella* and native microflora. The average daily air temperature from 4 April to 22 May 2023, in Riyadh, Saudi Arabia, was 28.3 °C (range: 20.3 to 34.9 °C). The *Salmonella* and native microflora analysis of 100 eggs was started by random selection of two eggs per plate. This was conducted to simulate selection of eggs by the consumer so that the results would be representative. The average weight ± standard deviation of the chicken eggs was 62.4 ± 8.6 g (range: 50 to 100 g).

Data for *Salmonella* serotype prevalence and number (growth units) for each egg were obtained using the PFARM whole sample enrichment (WSE), quantitative real-time polymerase chain reaction (qPCR), culture isolation, serotyping, and the Monte Carlo simulation method [14] with modifications to local resources. The chicken egg being analyzed was cracked using a sterilized knife inside a sterile Seward bag (BA6041/CLR closure, Seward, London, UK). Next, 400 mL of prewarmed (40 °C) buffered peptone water (BPW, CM0509, Oxoid, Basingstoke, UK) was added. The egg sample in BPW was then homogenized for one min at normal speed in a paddle-style blender. The WSE phase for enumeration of *Salmonella* was conducted by incubating the homogenized egg sample in BPW at 40 °C and 80 revolutions per min (rpm) for six hours.

After WSE for the enumeration of *Salmonella*, the egg sample in BPW was held overnight at 4 to 6 °C. The following day, 1 mL of the WSE was used for DNA extraction and enumeration of *Salmonella* by qPCR, and the rest of the WSE was incubated at 37 °C for an additional 18 to 24 h to complete the non-selective, pre-enrichment phase of the *Salmonella* isolation method for serotype prevalence.

A *Salmonella* Heidelberg isolated from a chicken egg was used to develop the standard curve for enumeration of *Salmonella* by WSE-qPCR. Stationary phase cells of the isolate were suspended in sterilized distilled water and adjusted to 0.5 McFarland. Serial dilutions (1:10) were made in BPW. An amount of 1 mL of each serial dilution was plated in 3M™ Petrifilms followed by incubation at 37 °C for 24 h to find the concentration of *Salmonella* Heidelberg in each serial dilution for calculation of the log_10_ dose inoculated into chicken egg samples for standard curve development.

To obtain data for standard curve development, 14 chicken egg samples were inoculated with 100 µL of serial dilutions from 10^−2^ to 10^−7^ followed by incubation for six hours at 40 °C and 80 rpm. After incubation, 1 mL of WSE was transferred to a 1.5 mL microcentrifuge tube for DNA extraction [19]. The WSE samples were centrifuged at 12,000 rpm for two minutes. The pellets were resuspended in 100 µL of PrepMan Ultra Sample Preparation Reagent (Applied Biosystem, Foster City, CA, USA) and then vortexed for 10 to 30 s. The vortexed samples were heated for ten minutes at 100 °C and then cooled for two minutes. Lastly, the samples were centrifuged at 12,000 rpm for two minutes and then 5 µL of the supernatant was transferred to clean tubes for qPCR analysis.

The qPCR Master mix was prepared using SureFast *Salmonella* ONE Kit (R-Biopharm, Darmstadt, Germany). The final reaction volume was 25 µL, which included 5 µL of the isolated DNA template. The qPCR reactions were run on an Applied Biosystems thermocycler with the following program: a cycle of DNA polymerase activation of five minutes at 95 °C followed by 45 amplification cycles of 15 s at 95 °C and 30 s at 60 °C for the annealing extension step. An internal amplification standard and positive and negative controls were used for quality control to ensure that the qPCR was reliable and duplicate technical replicates were run and found to be highly reproducible. The primary sequences of the primers used in the qPCR are not available because of proprietary reasons.

**Figure 2:**
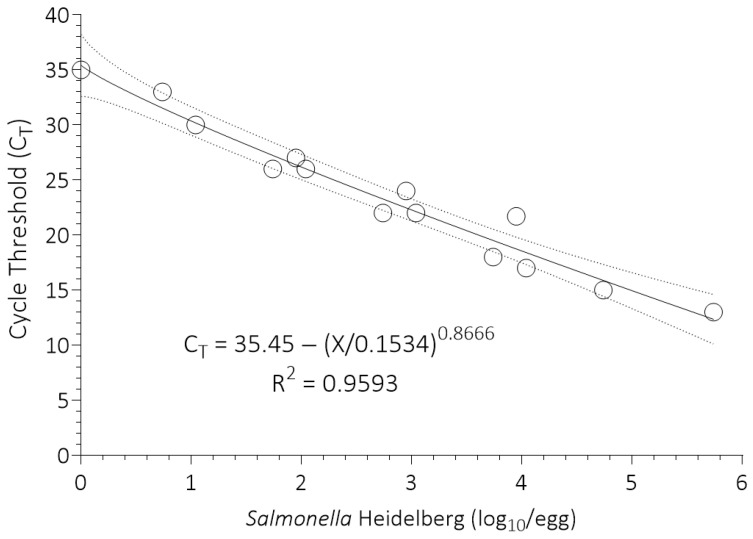
Standard curve for enumeration of *Salmonella* in chicken eggs. The R^2^ is the coefficient of determination for the fit of the Weibull model to the data. The dotted lines are the 95% confidence interval around the best-fit line (solid line). The X is the log_10_ dose of inoculated *Salmonella* Heidelberg.

Cycle threshold values were graphed as a function of the log_10_ dose of *Salmonella* Heidelberg inoculated into the egg samples (Figure 2). The data were fitted to the Weibull model using GraphPad Prism (version 10.3 for Windows, GraphPad Software, Boston, MA, USA, www.graphpad.com) to obtain the standard curve and the 95% confidence interval for enumeration of *Salmonella* in uninoculated chicken egg samples (*n* = 100), which were subjected to the same WSE-qPCR procedure but without the addition of *Salmonella* Heidelberg. The coefficient of determination (R^2^) and 95% confidence intervals, measures of the accuracy of the standard curve, and the range of enumeration (0 to 5.8 log_10_ growth units per chicken egg) are shown in Figure 2.

After the first six hours of WSE for the enumeration of *Salmonella*, the uninoculated chicken egg samples (*n* = 100) were incubated for an added 18 to 24 h at 37 °C for isolation of *Salmonella* serotypes as follows. One hundred microliters of the BPW incubate was transferred to ten mL of Rappaport–Vassiliadis Broth (RVB, CM0669, Oxoid) for selective enrichment at 42 °C for 24 h. Next, RVB incubate was spread plated on Xylose Lysine Deoxycholate (XLD, CM0469B, Oxoid) agar and Hektoen Enteric (HE, CM0419B, Oxoid) agar. Spread plates were incubated for 24 h at 37 °C. A colony from XLD (red colony with black center) or HE (green to blue-green colony with black center) that showed typical *Salmonella* morphology was picked and used to prepare a stock culture that was subsequently used for confirmation and serotyping.

Suspected *Salmonella* isolates were confirmed by MALDI-TOF [20]. In brief, 1 µL of a resuspended *Salmonella* colony was spread on a MALDI target plate and allowed to dry. Then 1 µL of α-cyano-4 hydroxycinnamic acid (Sigma Aldrich, St. Louis, MO, USA) was applied to the dried sample and allowed to co-crystalize. The target plate was then placed into the MALDI-TOF instrument (Bruker Daltonik GmbH, Bremen, Germany). The sample was ionized using a laser and the generated mass spectra was compared to the MALDI Biotyper reference database (Bruker Daltonik) for identification as *Salmonella*. All samples were blinded and analyzed in duplicate.

After MALDI-TOF identification as *Salmonella*, isolates were serotyped using the White–Kauffmann–Le Minor system of serum agglutination with antisera to somatic O and flagella H antigens [21]. The zoonotic potential of the isolated serotype was calculated using epidemiological data from the United States and the PFARM method [15]. It ranged from 0.1 (lowest zoonotic potential) to 5.0 (highest zoonotic potential).

To determine the level of native microflora associated with the chicken eggs, which was needed for PFARM simulation for the total aerobic count, 3M™ Petrifilms were utilized [22]. One milliliter of two dilutions (10^−1^ and 10^−2^) of each egg sample was plated on 3M™ Petrifilms sheets, which were then incubated at 30 °C for three days. After incubation, the colonies were counted, and the results were calculated. The average ± standard deviation of the total aerobic bacterial count per egg was 4.69 ± 0.47 log_10_ (range: 4.09 to 6.24 log_10_). The variability and uncertainty of these data were simulated in the PFARM with a PERT distribution (minimum = 4.09, mode = 4.11, and maximum = 6.24 log_10_ per egg).

A consumer survey in the PFARM that was designed and validated for chicken gizzards [15] was modified slightly (see spreadsheet S in the Supplementary File) and then used to collect data for home storage practices for chicken eggs (time, temperature), meal preparation practices (hygiene, time, temperature, egg doneness), food consumption behavior (portion size, side dishes, beverage, antacid), and consumer health and immunity (age, body mass index (obesity), preexisting health conditions (cancer, diabetes, pregnancy), and medications (corticosteroids, acid reflux, oral antibiotics). The survey was administered by SFDA personnel to 125 ordinary, untrained consumers of chicken eggs in Riyadh, Saudi Arabia. Responses fell in one of five risk categories: 1 = very low; 2 = low; 3 = moderate; 4 = high; and 5 = very high risk. The output of the survey was a 9-digit code that kept responses confidential and thus, written consent and Institutional Review Board (IRB) review were not required [23]. See informed consent and IRB statements below for additional details.

The 9-digit code was the only information needed to simulate the survey results in the PFARM. Socioeconomic status was not directly included in the survey, which was focused on confidentiality and on specific risk behaviors, some of which are associated with socioeconomic status. It is acknowledged that more survey results would improve the statistical power of the results and that the method of survey administration may have introduced some potential bias in the responses of the consumers.

### 2.2. Modeling Methods

The PFARM for *Salmonella* and chicken eggs in Riyadh, Saudi Arabia (Supplementary File), was created in an Excel notebook (Office 365, MicroSoft Corporation, Redmond, WA, USA) and was simulated with @Risk (version 8.2, Decision Tools Suite, Lumivero, Denver, CO, USA). Details of the modeling methods for simulating the initial contamination [14], illness dose [15], dose consumed [12], and consumer response [16] steps of the PFARM are provided in the cited studies. Here, only the modifications of the modeling methods are provided in detail.

The PFARM for *Salmonella* and chicken eggs consisted of 11 spreadsheets (!): (1) table of contents and flow diagram (C!); (2) consumer survey (S!); (3) data input (D!); (4) initial contamination at retail (1!); (5) microbial growth during home storage (2!); (6) microbial cross-contamination of kitchen fomites (3!); (7) microbial cross-contamination of lettuce (4!); (8) *Salmonella* growth on lettuce (5!); (9) microbial death and survival during cooking (6!); (10) *Salmonella* dose–response after consumption (7!); and (11) appendix (A!).

The contamination of chicken eggs at retail with native microflora including *Salmonella* was simulated in 1! of the PFARM [14]. In brief, data for native microflora, *Salmonella* serotype prevalence and number (growth units), and results (9-digit code) from the consumer surveys (*n* = 125) were entered in D! of the PFARM. A rare event modeling method was used to simulate the distribution of native microflora and *Salmonella* among noncontaminated and contaminated servings (1 egg) and portions (1 to 5 eggs) of chicken egg meals at retail. A DISCRETE distribution was used to simulate *Salmonella* serotype prevalence:

= RiskDiscrete({0,1,2,3,4},{93,4,1,1,1})

where the left bracket was the serotype code (0 = none, 1 = Cerro, 2 = Enteritidis, 3 = Stanley, 4 = Winston) and the right bracket was the corresponding prevalence. When the randomly sampled value from the DISCRETE distribution was 0, the egg was not contaminated with *Salmonella*; otherwise, it was contaminated with the randomly selected *Salmonella* serotype from the DISCRETE distribution.

If two to five eggs were consumed in the meal, it was possible that two or more serotypes could be present. In this case, a composite zoonotic potential score was calculated based on the zoonotic potential of the serotypes and their number (growth units). For example, if one egg was contaminated with 100 growth units of *Salmonella* Enteritidis with a zoonotic potential of 5.0 and another egg in the meal was contaminated with 100 growth units of *Salmonella* Cerro with a zoonotic potential of 0.4, the composite zoonotic potential for the egg meal would be [(100/200) × 5.0] + [(100/200) × 0.4] = 2.7.

The growth of native microflora and *Salmonella* during home storage of poultry food was not simulated in the earlier PFARM [12]. Therefore, it is fully described here and represents one of the novel aspects of this study.

The growth of native microflora and *Salmonella* in chicken eggs during home storage was simulated in 2! of the PFARM. DISCRETE (incident of the event or risk category from the consumer survey) and UNIFORM (variability and uncertainty of the extent of the event when it occurred) distributions were used in the rare event method (linking of the incidence and extent of the event using the IF logical statement in Excel) to simulate consumer survey data for home storage time and temperature. Also, UNIFORM and PERT distributions were used in the rare event method to simulate effects of time and temperature and their variability and uncertainty on yolk membrane depletion in the farm-to-retail pathway using the biphasic model of Latimer et al. [24] with modifications:

Log_10_ (YMDt) = 2.0872 − 0.04257 × T

where T was temperature (°C) and YMDt was yolk membrane depletion time in days. The output from this section in 2! of the PFARM was the proportion of yolk membrane integrity lost (pYMIL) in the farm-to-retail pathway. A prerequisite for growth of *Salmonella* in chicken eggs is the depletion of the yolk membrane integrity so that *Salmonella* can gain access to the nutrient rich environment of the yolk [24]. Thus, there is a need for this model and the growth models that follow.

The time for microbial growth (*t*_g_) during home storage was calculated from home storage time (*t*_hs_), proportion of yolk membrane integrity at retail (pYMIR), yolk membrane depletion time during home storage (YMDt_hs_), and lag time (Lt):

*t*_g_ = 0      IF *t*_hs_ − (YMDt_hs_ × pYMIR) − Lt ≤ 0

*t*_g_ = *t*_hs_ − (YMDt_hs_ × pYMIR) − Lt  IF *t*_hs_ − (YMDt_hs_ × pYMIR) − Lt > 0

where time was in hours and where growth of native microflora and *Salmonella* in the egg only occurred after depletion of yolk membrane integrity and lag time.

The time for growth was limited by the maximum microbial population supported by the egg, which was fixed at 9.79 log_10_ per egg and was equal to a microbial density of 8.0 log_10_ per gram of egg contents for spoilage [25] plus the log_10_ of egg weight, which was fixed at 62 g or 1.79 log_10_ g. Thus, once yolk membrane depletion and lag time was complete, the growth of native microflora and *Salmonella* started and then stopped when either native microflora or *Salmonella* reached the maximum population level of 9.79 log_10_ per egg or when the time for growth had expired. In other words, the Jameson effect [26] was simulated. This was performed using models that predicted the lag time (h) and growth rate (log_10_/h) of native microflora and *Salmonella* as a function of temperature.

The lag time and growth rate of the native microflora were simulated using the data of Ohkochi et al. [27] for the growth of native microflora in pasteurized eggs incubated at temperatures from 4.1 to 19.4 °C. The lag time (Lt) data were transformed to their log_10_ and fitted into a linear model, while the growth rate (GR) data were transformed to their square root and fitted into a linear model:

Log_10_ Lt = 2.89 − 0.1108T; R^2^ = 0.9980

√GR = 0.006621+ 0.02764T; R^2^ = 0.9884

where T was temperature (°C) and R^2^ was the coefficient of determination. The models were extrapolated to 30 °C, which was the maximum home storage temperature for the consumers surveyed in this study. Thus, the temperature thresholds for growth rate and lag phase were from 4 to 30 °C based on the results of the consumer survey. The models were coded into 2! of the PFARM. The lag time was the period before growth started and in which the bacteria were adjusting to their new environment, whereas the amount of growth after the lag phase depended on the growth rate, which was log-linear until stationary phase or until a bacterial concentration of 9.79 log_10_ per egg was reached. Thus, the need for both lag time and growth rate models.

The growth of *Salmonella* in chicken eggs was simulated using the lag time and growth rate models of Singh et al. [28] in liquid whole egg:

Lt = 3.51/GR

GR = 0      IF T < 6.1 °C or > 45.19 °C

GR = 0.0016 × (T − 6.1)^2^ × (1 − exp(0.2014 × (T − 45.19))) IF T ≥ 6.1 °C and ≤45.19 °C

where 3.51 was the physiological state parameter, T was temperature, 6.1 was the minimum growth temperature (°C), 45.19 was the maximum growth temperature (°C), and 0.0016 and 0.2014 were regression coefficients. The models were coded into 2! of the PFARM.

The cross-contamination of kitchen fomites (hands, surfaces, utensils…) with native microflora and *Salmonella* was simulated in 3! of the PFARM using a rare event and line of transfer method [12] with one modification. The risk categories for hygiene from the consumer survey were used to identify the corresponding UNIFORM distribution for microbial transfer rate in A! of the PFARM, which were 0.0 to 0.000161, 0.000161 to 0.000806, 0.000806 to 0.001613, 0.001613 to 0.008065, and 0.008065 to 0.016129 for risk categories 1, 2, 3, 4, and 5, respectively. These transfer rates were based on the assumptions that consumers contaminated kitchen fomites with 0 to 0.01, 0.01 to 0.05, 0.05 to 0.1, 0.1 to 0.5, and 0.5 to 1 g of chicken egg contents (62 g) for risk categories 1, 2, 3, 4, and 5, respectively. In addition, the transfer rates and cross-contamination pathways simulated were based on experiments with chicken meat [29,30,31] and eggs [32].

The cross-contamination of lettuce with native microflora and *Salmonella* was simulated in 4! of the PFARM using a rare event and line of transfer method [12] with one modification. A computational limitation of the line of transfer method in the PFARM was that only 10,000 rows were used per egg to simulate transfer of individual *Salmonella* growth units from raw eggs to kitchen fomites to lettuce. Thus, to simulate potential transfer of greater than 10,000 growth units of *Salmonella* per raw egg after growth during home storage, it was assumed that the first 10,000 growth units represented mother cells and daughter cells growing alone whereas all other cells ≥ 10,001 growth units were daughter cells growing in a transfer cluster with their mother cell.

The line of transfer method simulates *Salmonella* as a minority member of the native microflora [12]. To do this, the PFARM randomly assigns individual growth units of *Salmonella* to a position in the line of transfer. Thus, if an egg is contaminated with 1000 cells of native microflora including one growth unit of *Salmonella*, the location of the *Salmonella* growth unit can, by random chance, be anywhere from position 1 to 1000. If 100 cells of native microflora are transferred to kitchen fomites or lettuce, and the *Salmonella* growth unit is assigned, by random chance, to a position from 1 to 100, it is transferred; otherwise, it is not.

The growth of *Salmonella* on lettuce after cross-contamination was simulated in 5! of the PFARM. Here, consumer survey results for meal preparation time and kitchen temperature were used in a validated model for growth of a chicken isolate of *Salmonella* Newport on Romaine lettuce held at 16 to 40 °C for 0 to 8 h [33]. The PFARM growth predictions for native microflora in eggs and *Salmonella* in eggs and on lettuce were not validated by correlation analysis because it was not technically possible to collect the needed validation data. Specifically, it was not possible to recreate the simulated conditions in the PFARM in challenge studies with eggs and lettuce because it was not possible to know the levels of native microflora and levels and serotype of *Salmonella* in an egg before conducting a storage trial with it. Also, it was not possible to collect enough data for a complete and not confounded correlation analysis. In fact, one replicate simulation of the current PFARM involved 12,500 (12,500 egg meals × 100%) unique combinations of conditions for potential growth of native microflora in eggs and 875 (12,500 egg meals × 7%) unique combinations of conditions for potential growth of *Salmonella* in eggs. Thus, the best that could be done was to use the best available predictive models for growth of native microflora in eggs and *Salmonella* in eggs and on lettuce, as was conducted in the current study.

The death and survival of native microflora and *Salmonella* during cooking of the eggs was simulated in 6! of the PFARM using consumer survey data for egg doneness and a line of death method that was like the line of transfer method [12]. The consumer survey asked about fried egg doneness. There were five choices from very low to very high risk: 1 = over hard; 2 = over medium; 3 = over easy; 4 = sunny side up; and 5 = raw. These categories corresponded to UNIFORM distributions in A! of the PFARM for the log_10_ reduction in native microflora including *Salmonella* per gram of egg contents. They were 7 to 12, 4.66 to 7, 2.33 to 4.66, 0 to 2.33, and 0 log_10_ reduction for risk categories 1, 2, 3, 4, and 5, respectively.

The simulation results for *Salmonella* dose consumed from 6! in the PFARM and the consumer survey results for food consumption behavior and consumer health and immunity were used in 7! of the PFARM to simulate illness dose [15] and consumer response [16]. Here, the ratio of *Salmonella* dose consumed (DC) to illness dose (ID) was used to simulate the consumer responses (health outcomes) of no response, infection, illness, hospitalization, and death (Figure 1). One hundred and twenty-eight PERT distributions for illness dose were used to simulate the variability and uncertainty of the dose–response interaction between *Salmonella* serotype (zoonotic potential), food consumption behavior (buffering capacity of the egg meal), and consumer health and immunity (resistance to salmonellosis).

The PFARM for *Salmonella* and chicken eggs was simulated with settings of Latin Hypercube sampling, Mersenne Twister generator, initial seeds of 1, 8, 11, 28, 32, 46, 52, and 69, and 12,500 iterations (100 egg meals/consumer). The average egg consumption from the consumer survey was 2.384 eggs per meal for 29,800 eggs per replicate simulation. Individual consumer survey results (*n* = 125) were simulated using a UNIFORMINT distribution in D! of the PFARM. This was performed to avoid simulating survey results that did not occur. Simulation results were exported to Excel, where the PIVOT TABLE function was used to tabulate the cases of no response, infection, illness, hospitalization, and death per consumer and overall. A sensitivity analysis was then conducted to identify potential risk factors for salmonellosis using XY scatter plots of selected model outputs versus health outcomes with a focus on correlation with cases of salmonellosis.

One hundred egg meals (iterations) were simulated per consumer (*n* = 125) in each replicate simulation (initial seed) to provide the right amount of data (*n* = 12,500) for clarity of presentation of scatter plots of model outputs. Also, eight replicate simulations (initial seeds) were run to provide the right amount of data (*n* = 100,000) for comparison of the predicted rate of salmonellosis to epidemiological data for salmonellosis, which is often expressed per 100,000 people [2]. The zoonotic potential of *Salmonella* [15] and the consumer responses of no response, infection, illness, hospitalization, and death [16] are based on epidemiological data from the United States.

## 3. Results and Discussion

### 3.1. Data Collection Results and Discussion

The standard curve for enumeration of *Salmonella* in chicken eggs at retail in Riyadh, Saudi Arabia, is shown in Figure 2. The range of enumeration was from 0 to 5.8 log_10_ growth units of *Salmonella* per egg. Results were expressed in growth units because this is a growth-based assay that captures the physiological state of the *Salmonella* and effects of the native microflora on their growth potential, which is important for accurate risk assessment [14]. In contrast, the most probable number method used by other risk assessors [13,34,35] does not capture the growth potential of *Salmonella* as influenced by their physiological state and interaction with native microflora and thus, is less accurate [11,14].

A similar WSE-qPCR method was used by Jakociune et al. [36] to develop standard curves for enumeration of *Salmonella* in chicken eggs. They stressed the importance of developing standard curves for enumeration that are matrix specific, like the one used in this study. However, they did not report any data for *Salmonella* contamination of chicken eggs. In contrast, in the present study, the interpolation function of the curve-fitting software was used to convert cycle threshold values for *Salmonella* contaminated chicken eggs to log_10_ growth units ± their 95% confidence interval (Table 1). Cycle threshold values were obtained for five of the seven chicken egg samples that were positive for *Salmonella* by culture methods. They ranged from 22 to 35, which corresponded to 3.077 to 0.061 log_10_ growth units per egg, respectively.

The two samples that tested negative in the WSE-qPCR assay but positive in the *Salmonella* isolation procedure were assumed to have 0 log_10_ growth units per egg because their growth was slower under the same conditions as a single growth unit of *Salmonella* Heidelberg, which was used to develop the standard curve for enumeration and was grown under optimal conditions. The slower growth of the *Salmonella* in these two egg samples could be due to their physiological state (sublethal injury) and (or) interaction with the native microflora (microbial competition). Thus, the sensitivity of the WSE-qPCR method is one growth unit of healthy *Salmonella* per the size of sample analyzed.

Variability and uncertainty of model inputs were simulated together throughout the model using probability distributions and the rare event modeling method. On the other hand, different initial seeds were used to assess the variability and uncertainty of model outputs among replicate simulations due to the rare, random, variable, and uncertain nature of multiple events and model inputs in the risk pathways.

The variability and uncertainty of the enumeration results for *Salmonella* were simulated in D! of the PFARM using a PERT distribution with a minimum of 0, mode of 1.58, and maximum of 3.34 log_10_ growth units per egg. The mode and range of log_10_ growth units per egg are like the mode and range of log_10_ growth units per size of sample analyzed in other studies that used the WSE-qPCR method to enumerate *Salmonella* in poultry [14,17,18] and pork [37,38] meat samples.

The short WSE (6 to 8 h) and qPCR (4 to 5 h) steps in this method make it fit-for-purpose for test-and-hold programs in the food industry [36]. Also, when combined with Monte Carlo simulation, like in this study, *Salmonella* data collected with one sample size (1 egg) can be used to obtain *Salmonella* data for larger samples sizes (2 to 5 eggs) at no extra cost [14,39].

The predominant *Salmonella* serotype isolated from chicken eggs (Table 1) was Cerro (*n* = 4) followed by Enteritidis (*n* = 1), Stanley (*n* = 1), and Winston (*n* = 1). The zoonotic potential was 0.4 (range: 0.2 to 0.5) for Cerro (very low risk), 5.0 (range: 4.9 to 5.0) for Enteritidis (very high risk), 2.2 (range: 1.6 to 2.6) for Stanley (moderate risk), and 0.1 (range: 0.1 to 0.1) for Winston (very low risk). The variability and uncertainty of zoonotic potential was simulated in D! of the PFARM using PERT (minimum, mode, maximum) distributions that captured strain variation. The zoonotic potential captures differences in the ability of *Salmonella* serotypes to persist in the environment, animal host, and to cause disease in humans, and thus is a more complete predictor of salmonellosis than virulence, which only considers differences in ability of serotypes to cause disease in humans [15].

Archer et al. [40] examined the eggshell (*n* = 384) and egg contents (*n* = 384) of chicken eggs obtained at retail markets in Ghana for *Salmonella*. They isolated *Salmonella* from both eggshells (*n* = 19; 4.9%) and egg contents (*n* = 7; 1.8%). The overall *Salmonella* prevalence of 5.5% was like the overall *Salmonella* prevalence of 7% in the current study. The isolated serotypes of *Salmonella* were Ajiobo (*n* = 1), Chester (*n* = 6), Hader (*n* = 7), Enteritidis (*n* = 2); and I 4:b:- (*n* = 8) with only Enteritidis being isolated in the current study.

Tessema et al. [41] examined the prevalence of *Salmonella* isolated from the eggshell and egg contents of chicken eggs (*n* = 384) obtained from a farm in Chile. They isolated *Salmonella* from both eggshells (*n* = 9; 2.3%) and egg contents (*n* = 2; 0.5%) for an overall prevalence of 2.8%, which was more like that in the study of Archer et al. [40] (5.5%) than in the current study (7%). Like the study of Archer et al. [40], *Salmonella* prevalence was higher for eggshell than egg contents. Thus, including the eggshell in the analysis for *Salmonella* in the present study likely resulted in a fail-safe prediction of the risk and severity of salmonellosis.

Results of the consumer survey are summarized in Table 2 as a function of the number of responses per risk category. The footnotes show the ranges for the results in each risk category. The mean risk category per query ranged from 1.1 for meal preparation time to 3.7 for home storage time where 1 was very low risk and 5 was very high risk. The overall risk among consumers (*n* = 125) for the items surveyed was 2.2 ± 0.8 (low). The consumer survey results are discussed in more detail in the simulation results section below.

### 3.2. Simulation Results and Discussion

**Figure 3:**
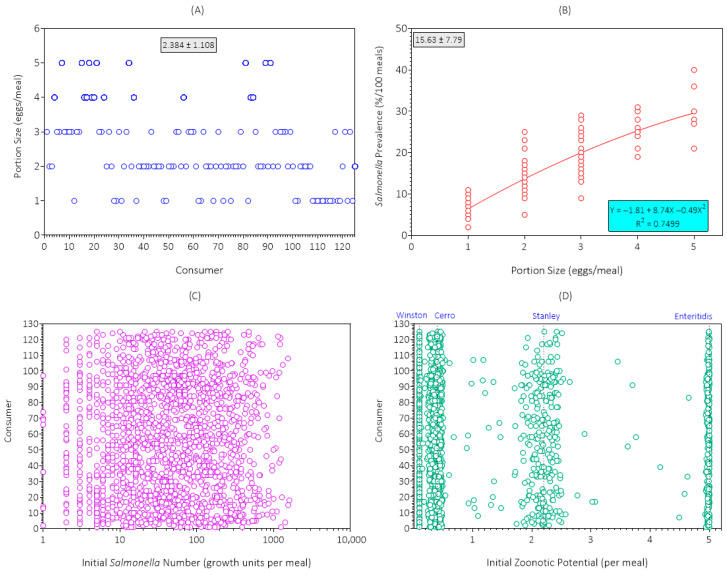
Simulation results for (**A**) portion size; (**B**) *Salmonella* prevalence; (**C**) *Salmonella* number (growth units); and (**D**) *Salmonella* serotype (zoonotic potential) in egg meals at retail in the first replicate simulation (initial seed = 1) of the Poultry Food Assess Risk Model for *Salmonella* and chicken eggs in Riyadh, Saudi Arabia.

The first step was to simulate contamination of *Salmonella* among egg meals at retail for the 125 consumers (Figure 3). The simulation results for portion size for the first replicate simulation (initial seed = 1; Figure 3A) indicated that the average number of eggs consumed per meal was 2.384 (range: 1 to 5 eggs), which agreed with results of the consumer survey (Table 2).

The simulation results for *Salmonella* prevalence as a function of egg consumption (portion size) at retail for the first replicate simulation are shown in Figure 3B. The mean *Salmonella* prevalence at retail was 15.63% per 2.384 eggs. The average predicted prevalence of *Salmonella* rose nonlinearly from 6.44% per 1 egg to 29.4% per 5 eggs, showing significant variability and uncertainty within portion sizes due to the random choice of eggs from the lot.

*Salmonella* prevalence of chicken eggs (*n* = 5548) in China was reported as 0.5% per 6 eggs with a predominance of serotype Enteritidis [42]. *Salmonella* Enteritidis can contaminate chicken eggs during egg formation, whereas other *Salmonella* like Cerro, Stanley, and Winston, which were isolated from chicken eggs in this study, can contaminate chicken eggs through fecal contamination and egg penetration [43]. Assuming these scenarios of *Salmonella* transmission to chicken eggs in the present study, if the shell had not been included in the current *Salmonella* test samples, the observed *Salmonella* prevalence could have been 1% instead of 7% per egg and thus be in closer agreement with the results from China. Although not consumed, the shell was included in the analysis to increase the chances of *Salmonella* detection for evaluating the PFARM in this study, and to provide a fail-safe prediction of the risk and severity of salmonellosis. Other factors such as storage practices, supply chain variability, and regional farming conditions could explain the difference in *Salmonella* serotype prevalence among studies.

The simulation results for *Salmonella* number (growth units) for the first replicate simulation are shown in Figure 3C. The distribution of *Salmonella* growth units among egg meals at retail for each consumer was variable and uncertain (range: 1 to 1602) because the eggs in each meal were selected randomly from the same lot and because most eggs (93%) in the lot were not contaminated with *Salmonella*. Differences in portion size (eggs/meal; Figure 3A) also explained the variability and uncertainty among consumers.

The simulation results for the distribution of zoonotic potential of *Salmonella* serotypes among egg meals at retail for each consumer for the first replicate simulation are shown in Figure 3D. The predominant serotype was Cerro, and the highest risk serotype was Enteritidis. Egg meals with multiple serotypes had zoonotic potentials that fell between individual serotypes. The distribution of serotypes was similar among consumers because eggs were from the same lot and slightly different because of differences in portion size (eggs/meal) among consumers (Figure 3A). The zoonotic potential of the egg meals at retail was variable and uncertain for individual consumers because of the random selection of eggs from the lot.

The second step in the PFARM simulated illness dose [15], which is the minimum dose of *Salmonella* consumed that results in an illness or case of salmonellosis for the simulated *Salmonella* serotype, egg meal, and consumer interaction. The zoonotic potential of the *Salmonella* serotype(s) consumed, the buffering ability of the egg meal, and the health and immunity of the consumer interact to decide the illness dose.

**Figure 4:**
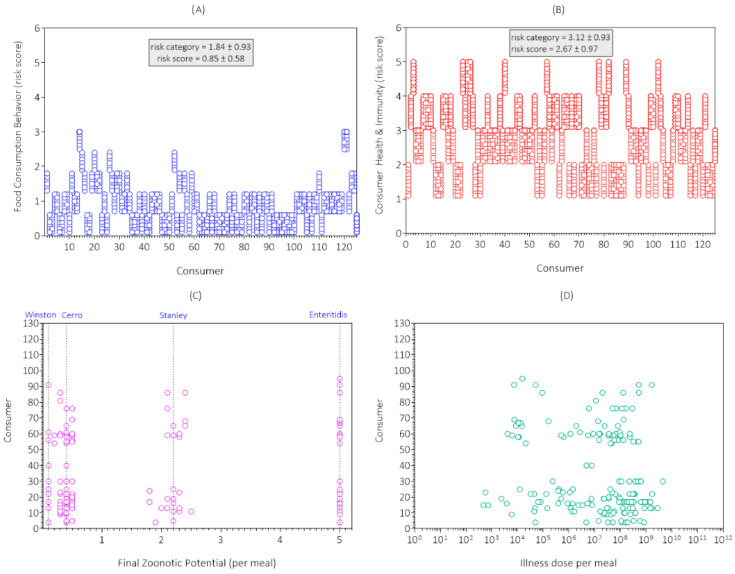
Simulation results for (**A**) food consumption behavior; (**B**) consumer health and immunity; (**C**) *Salmonella* serotype (zoonotic potential); and (**D**) illness dose in egg meals at consumption in the first replicate simulation (initial seed = 1) of the Poultry Food Assess Risk Model for *Salmonella* and chicken eggs in Riyadh, Saudi Arabia.

The simulation results for food consumption behavior in the first replicate simulation are shown in Figure 4A. The mean risk category for food consumption behavior (buffering capacity of the egg meal) was 1.84, which agreed with the consumer survey results (Table 2). The mean risk score on a scale from 0.1 (very low) to 3.0 (very high) was 0.88 (low). Most consumers (81.6%) ate egg meals with low buffering capacity, which would lower their risk of salmonellosis by making it harder for *Salmonella* to survive passage through the gastric acidity of the stomach [44,45,46]. UNIFORM distributions in A! of the PFARM were used to simulate the variability and uncertainty of the buffering ability of the meal within a risk category, as demonstrated in Figure 4A.

The simulation results for consumer health and immunity for the first replicate simulation are shown in Figure 4B. The mean risk category for consumer health and immunity was 3.12, which agreed with the consumer survey results (Table 2). The mean risk score on a scale from 0.1 (very low) to 5.0 (very high) was 2.67 (moderate). The percentage of high-risk consumers was 34.4% (43/125). UNIFORM distributions in A! of the PFARM were used to simulate the variability and uncertainty of consumer health and immunity within a risk category, as demonstrated in Figure 4B.

The simulation results for zoonotic potential of the *Salmonella* serotype(s) consumed in the egg meals for the first replicate simulation are shown in Figure 4C. In contrast to the zoonotic potential of *Salmonella* serotypes in egg meals at retail (Figure 3D), fewer meals were contaminated with *Salmonella* at consumption and few if any were contaminated with multiple serotypes (intermediate zoonotic potentials). This was because cooking removed *Salmonella* from many eggs that were contaminated at retail.

The zoonotic potential of egg meals that were still contaminated with *Salmonella* at consumption was variable and uncertain between and within consumers (Figure 4C) because it was by random chance which eggs were selected from the lot, which eggs were contaminated with *Salmonella* at retail, which serotype(s) were present, which portions of lettuce were cross-contaminated, and which eggs were undercooked.

The zoonotic potential of the egg meal at consumption (Figure 4C) was added to the risk scores for food consumption behavior (Figure 4A) and consumer health and immunity (Figure 4B) to obtain a disease triangle score, which was used to calculate the input values (minimum, mode, maximum) for a PERT distribution for illness dose [15]. This distribution was randomly sampled to obtain the illness dose for the simulated *Salmonella* serotype, egg meal, and consumer interaction (Figure 4D). Illness dose could only be calculated for egg meals that resulted in exposure to *Salmonella*.

The illness dose for an individual consumer was variable and uncertain because the components of illness dose (zoonotic potential, meal buffering ability, consumer resistance) were variable and uncertain. In fact, the illness dose for an individual consumer often spans multiple log_10_ doses. For example, the illness dose for consumer 30 in Figure 4D ranged from 10^5.1^ to 10^9.7^. Consumers who handled and prepared eggs safely were not exposed to *Salmonella*. Thus, they did not have an illness dose.

The third step or dose consumed step [12] of the PFARM simulated how the distribution of *Salmonella* among egg meals changed from retail to consumption. First, growth of native microflora including *Salmonella* in eggs during home storage was simulated in 2! of the PFARM. This required data for home storage time and temperature from the consumer survey (Table 2).

**Figure 5:**
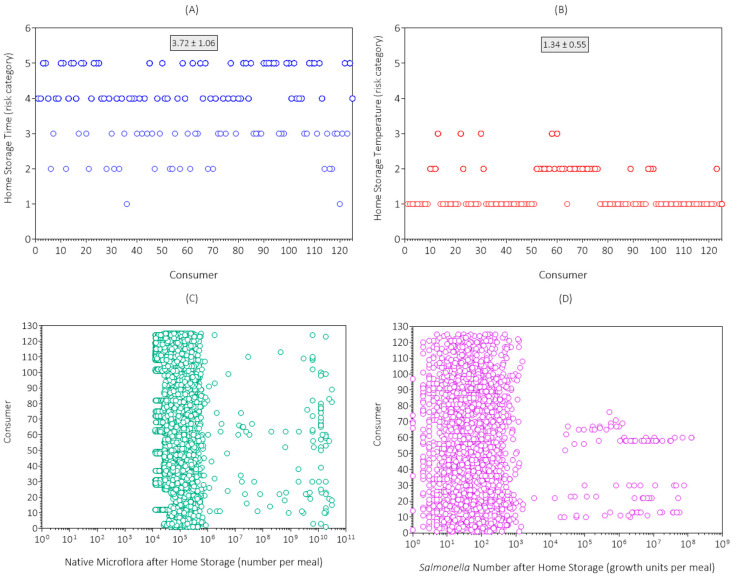
Simulation results for (**A**) home storage time; (**B**) home storage temperature; (**C**) native microflora; and (**D**) *Salmonella* number (growth units) in egg meals after home storage in the first replicate simulation (initial seed = 1) of the Poultry Food Assess Risk Model for *Salmonella* and chicken eggs in Riyadh, Saudi Arabia.

The simulation results for home storage time from the first replicate simulation are shown in Figure 5A. The mean risk category for home storage time was 3.72 (high risk), which agreed with the consumer survey results (Table 2). Most consumers (85.6%) stored eggs from 3 to 21 days.

The simulation results for home storage temperature from the first replicate simulation are shown in Figure 5B. The mean risk category for home storage temperature was 1.34 (very low risk), which agreed with the consumer survey results (Table 2). Most consumers (70.4%) stored their eggs at < 8 °C.

The simulation results for growth of native microflora during home storage of eggs in the first replicate simulation are shown in Figure 5C. Here, 5.3% of the egg meals had levels of native microflora (10^6.26^ to 10^10.2^) after home storage that were above the range (10^4.0^ to 10^6.25^) at retail. Thus, in a small proportion of the egg meals, the home storage time and temperature supported complete loss of yolk membrane integrity, exhaustion of lag time, and microbial growth. The level of native microflora among egg meals after home storage was variable and uncertain and was more variable and uncertain for consumers that stored their eggs for times and temperatures that supported microbial growth.

Although the maximum microbial number per egg was limited to 10^9.79^, some egg meals exceeded this level because of the consumption of more than one egg in the meal. For example, if a consumer ate five eggs and they all contained the maximum level of native microflora after home storage, the level of native microflora would be 10^10.5^ for that meal.

The simulation results for growth of *Salmonella* in eggs during home storage in the first replicate simulation are presented in Figure 5D. Here, 0.792% of egg meals had levels of *Salmonella* (10^3.4^ to 10^8.1^) after home storage that were above the range (10^0^ to 10^3.3^) at retail. The incidence of *Salmonella* growth among egg meals was less than the incidence of native microflora growth (Figure 5C) because most eggs subjected to conditions supporting microbial growth were not contaminated with *Salmonella*. The growth units of *Salmonella* among egg meals after home storage was variable and uncertain and was more variable and uncertain for consumers that stored their eggs under conditions of time and temperature that supported microbial growth.

Two exposure pathways were simulated. First, from raw eggs to kitchen fomites to lettuce to the consumer. Second, from eggs that were not fried or fried to various levels of doneness (sunny side up, over easy, over medium, or over hard) to the consumer. To simulate these exposure pathways, data for consumer hygiene during meal preparation and egg doneness were obtained from the consumer survey, respectively (Table 2).

**Figure 6:**
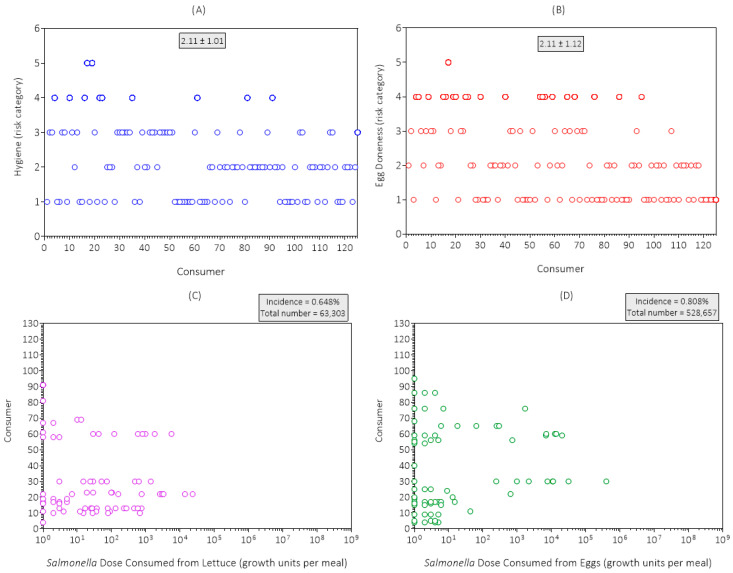
Simulation results for (**A**) hygiene; (**B**) egg doneness; (**C**) *Salmonella* dose (growth units) from lettuce; and (**D**) *Salmonella* dose (growth units) from eggs at consumption in the first replicate simulation (initial seed = 1) of the Poultry Food Assess Risk Model for *Salmonella* and chicken eggs in Riyadh, Saudi Arabia.

The simulation results for hygiene for the first replicate simulation are shown in Figure 6A. The mean risk category for hygiene was 2.11 (low risk), which agreed with the consumer survey results (Table 2). The risk category for hygiene was used to simulate cross-contamination of kitchen fomites and then lettuce with native microflora including *Salmonella*.

The simulation results for egg doneness for the first replicate simulation are shown in Figure 6B. The mean risk category for egg doneness was 2.11 (low risk), which agreed with consumer survey results (Table 2). One consumer ate raw eggs (risk category 5) while 39.2% of consumers ate completely cooked eggs (over hard).

The simulation results for doses (growth units) of *Salmonella* consumed from lettuce in the first replicate simulation are presented in Figure 6C. The incidence of *Salmonella* exposure from lettuce in egg meals was 0.648% and the total dose of *Salmonella* consumed from lettuce in egg meals was 63,303 growth units per 12,500 egg meals. Most consumers were not exposed to *Salmonella* from cross-contamination of lettuce in their egg meals. Exposure to *Salmonella* from lettuce in egg meals was variable and uncertain because the components of this exposure pathway (*Salmonella* growth units at retail and after home storage, hygiene during meal preparation, and portion size) were variable and uncertain.

The simulation results for the dose (growth units) of *Salmonella* consumed from eggs in the first replicate simulation are shown in Figure 6D. The incidence of *Salmonella* exposure from eggs in egg meals was 0.808% and the total dose of *Salmonella* consumed was 528,657 growth units per 12,500 egg meals. Thus, the predominant exposure pathway for consumers in the simulated scenario was undercooked eggs.

**Figure 7:**
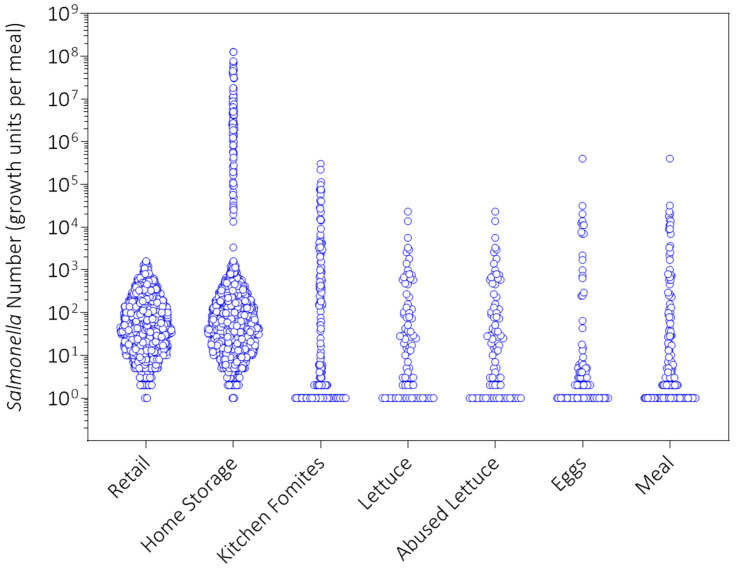
Simulation results for *Salmonella* number (growth units) in egg meals from retail to consumption in the first replicate simulation (initial seed = 1) of the Poultry Food Assess Risk Model for *Salmonella* and chicken eggs in Riyadh, Saudi Arabia.

No growth of *Salmonella* on lettuce after cross-contamination was seen in the first replicate simulation (Figure 7). Although kitchen temperatures in the surveyed population were high enough to support the growth of *Salmonella* on lettuce (Table 2), all meal preparation times were too short (<2 h) for *Salmonella* to exit the lag phase and grow on lettuce. In fact, the lag time for *Salmonella* on lettuce ranges from > 8 h at 16 °C to about 2 h at 30 °C [33], which was the maximum temperature in the kitchens of the surveyed consumers (Table 2).

The simulation results in Figure 7 also show that a consumer can be exposed to *Salmonella* from undercooked eggs and from cross-contaminated lettuce in the same egg meal as the incidence of *Salmonella* exposure from both pathways (meal) was 1.3%, which was higher than that from lettuce (0.648% in Figure 6C) or eggs (0.808% in Figure 6D) alone.

The fourth step in the PFARM simulated consumer response [16] using the ratio of *Salmonella* dose consumed to illness dose (Figure 1). The consumer responses (health outcomes) simulated were no response, infection, illness, hospitalization, and death. No response occurred when the ingested dose of *Salmonella* died from exposure to gastric acid before being able to reproduce in the gastrointestinal tract resulting in no shedding, no antibody response, and no symptoms of salmonellosis. Infection occurred when all or part of the ingested dose of *Salmonella* survived passage through the gastric acidity of the stomach and survived the host immune response resulting in fecal shedding, antibody response, but no symptoms of salmonellosis. Illness occurred when all or part of the ingested dose of *Salmonella* survived passage through the gastric acidity of the stomach and survived the host immune response resulting in fecal shedding, antibody response, and mild symptoms of salmonellosis. Hospitalization or death occurred when all or part of the ingested dose of *Salmonella* survived passage through the gastric acidity of the stomach and survived the host immune response resulting in fecal shedding, antibody response, and severe (hospitalization) and fatal (death) symptoms of salmonellosis including septicemia.

**Figure 8:**
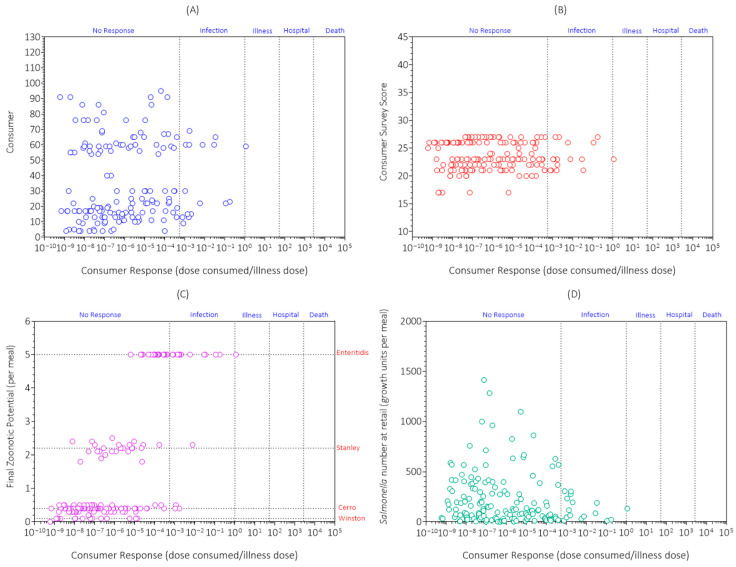
Simulation results for consumer response sorted by (**A**) consumer; (**B**) consumer survey score; (**C**) *Salmonella* serotype (zoonotic potential) at consumption; and (**D**) *Salmonella* number (growth units) at retail in egg meals in the first replicate simulation (initial seed = 1) of the Poultry Food Assess Risk Model for *Salmonella* and chicken eggs in Riyadh, Saudi Arabia.

The simulation results for consumer response sorted by consumer for the first replicate simulation are shown in Figure 8A. The consumer response was variable and uncertain between and within consumers; it ranged from 10^−10^ to 10^0.1^. Most egg meals resulted in no exposure (*n* = 12,337) to *Salmonella* followed by no response (*n* = 147), infection (*n* = 15), and illness (*n* = 1) with no cases of hospitalization or death when exposed to *Salmonella* (Table 3). Thus, salmonellosis (illness, hospitalization, death) from the examined and simulated chicken eggs and consumers was a rare event.

The simulation results for consumer response sorted by consumer survey score for the first replicate simulation are shown in Figure 8B. The consumer survey score was the sum of risk categories for the nine queries (Table 2) with a possible range from 9 to 45. However, in the consumer group surveyed, it only ranged from 12 to 27 with a mean of 19.6 ± 3.2. There was no correlation (Pearson r = 0.0055; *p* = 0.472; *n* = 125) between consumer survey score and consumer response. Thus, the consumer survey score was not a good predictor of chicken egg safety. This was because it did not consider all the risk factors for salmonellosis and how they interact. Importantly, it did not consider *Salmonella* serotype prevalence, zoonotic potential, and number (growth units) at consumption. Thus, it is important to simulate the survey results in the PFARM to properly assess the risk and severity of salmonellosis for the chicken egg consumers being studied.

The simulation results for consumer response sorted by zoonotic potential of *Salmonella* serotypes at consumption in the first replicate simulation are shown in Figure 8C. There was a positive correlation (Pearson r = 0.19; *p* = 0.0153; *n* = 163) between final zoonotic potential and consumer response. Within a serotype, consumer response was variable and uncertain. This was because the components of consumer response (*Salmonella* dose consumed and its components, and illness dose and its components) were variable and uncertain. Most exposures to the high-risk serotype Enteritidis did not cause illness indicating that other risk factors were involved. This finding and conclusion agrees with an earlier PFARM study with chicken gizzards [16] that indicated a case of salmonellosis only occurred when by random chance multiple risk factors occurred at the same time.

The simulation results for consumer response sorted by *Salmonella* number (growth units) in egg meals at retail are shown in Figure 8D. There was no correlation (Pearson r = −0.043; *P* = 0.583; *n* = 163) between the number (growth units) of *Salmonella* at retail and consumer response. In addition, all cases of infection and illness were associated with numbers (growth units) of *Salmonella* at retail that were below 300 and occurred most often. Thus, using final product test results for *Salmonella* to target the most contaminated eggs at retail would not reduce the risk and severity of salmonellosis. This is because it was by random chance which eggs were contaminated with serotype Enteritidis, which eggs were stored for times and temperatures that supported microbial growth, which eggs were handled in a careless manner during meal preparation resulting in cross-contamination of lettuce, which eggs were undercooked, which eggs were consumed with a high buffering capacity meal, and which eggs were consumed by someone at high risk for salmonellosis. This finding and conclusion agrees with other PFARM studies reviewed in Oscar [11,16]. However, Kim et al. [13] reached a different conclusion. Namely, that the most highly contaminated chicken parts with high-risk serotypes at final product testing pose the highest risk of salmonellosis. However, they did not consider growth of *Salmonella* on the chicken parts after final product testing, and they did not simulate buffering capacity of the meal, and consumer health and immunity in their dose–response model [11,16].

Table 3 shows a summary of the results for the eight replicate simulations of the PFARM for *Salmonella* and chicken eggs in Riyadh, Saudi Arabia. A total of 100,000 egg meals were simulated (800 egg meals per consumer). Most egg meals (98.73%) did not result in exposure to *Salmonella*. The cases of no response, infection, illness, hospitalization, and death were 1180, 88, 2, 0, and 0, respectively. Thus, the predicted cases of salmonellosis (illness, hospitalization, death) were 2 per 100,000 egg meals or consumers.

The finding of only two cases per 100,000 egg meals does not contradict the relatively high prevalence (7%) of *Salmonella* contamination at final product testing because it has been shown that there is no relationship between *Salmonella* prevalence at final product testing and cases of salmonellosis per lot of poultry food [11,16,18]. This is because many important risk factors for salmonellosis occur after final product testing, such as temperature abuse, undercooking, cross-contamination of ready-to-eat food, buffering capacity of the meal, and consumer meal preparation practices, eating habits, health, and immunity. To illustrate further how the current PFARM considers these mitigating factors, the details of the two observed cases of salmonellosis will now be examined. In addition, these examples will show how the PFARM works. In other words, how it considers all the mitigating factors in its prediction of consumer exposure and response to *Salmonella* in the simulated egg meal.

The risk factor combination that resulted in the two predicted cases of salmonellosis in Table 3 were identified from the simulation results and are presented in Table 4 for the two egg meals. In the first case of salmonellosis, consumer 59 ate three sunny side up eggs with a dose of 6896 growth units of *Salmonella* Enteritidis. At retail, the three eggs had 132 growth units of *Salmonella* Enteritidis and a yolk membrane integrity loss of 62% in the farm-to-retail pathway. The eggs were stored at home for 7 to 14 days at ≥8 to <20 °C resulting in growth of *Salmonella* Enteritidis to 1.3 × 10^6^ growth units. During meal preparation 650 growth units of *Salmonella* Enteritidis were transferred to kitchen fomites. However, no growth units of *Salmonella* Enteritidis were transferred to lettuce. The zoonotic potential of *Salmonella* Enteritidis was 5.0, the buffering capacity of the egg meal was moderate risk (food consumption behavior score = 1.8) and the consumers’ resistance to salmonellosis was moderate risk (consumer health and immunity score = 2.8). This resulted in a disease triangle score of 9.4, an illness dose of 6108, and consumer response of mild illness (DC/ID = 6896/6106 = 1.13). Thus, the risk factors that explain this case of mild salmonellosis were home storage of eggs at a time and temperature that supported microbial growth, undercooking of the eggs, and the random selection of eggs from the retail market that were contaminated with serotype Enteritidis.

The other case of salmonellosis involved consumer 22. In this case, the ratio of *Salmonella* dose consumed (100,940 growth units) to illness dose (84,883) was 1.19, which was a mild case of salmonellosis. At retail, the egg meal had 4283 growth units of *Salmonella* Enteritidis and the yolk membrane integrity loss was 40% in the farm-to-retail pathway. After home storage at temperatures from ≥20 to <30 °C for 7 to 14 days, the egg meal was contaminated with 1.8 × 10^9^ growth units of *Salmonella* Enteritidis. The consumer had poor hygiene during meal preparation resulting in transfer of 931,183 growth units of *Salmonella* Enteritidis to kitchen fomites followed by transfer of 100,940 growth units of *Salmonella* Enteritidis to lettuce. Meal preparation time was <1 h so the *Salmonella* Enteritidis on the lettuce did not grow. This consumer ate three over easy eggs that were undercooked resulting in survival of 279,895 cells of native microflora but none of them were *Salmonella* Enteritidis because by random chance they were not in the undercooked part of the eggs. The zoonotic potential of *Salmonella* Enteritidis was 5.0, the buffering capacity of the egg meal was moderate risk (food consumption behavior score = 1.8) and the consumers’ resistance to salmonellosis was high (consumer health and immunity score = 1.2). The disease triangle score was 7.9, the illness dose was 84,833, and the consumer response was a mild illness (DC/ID = 100,940/84,833 = 1.19). Thus, the risk factors for this mild case of salmonellosis were home storage of eggs for a time and temperature that resulted in microbial growth, poor hygiene during meal preparation that resulted in substantial cross-contamination of lettuce, and the random selection of eggs that were contaminated with *Salmonella* Enteritidis at retail.

Although these two consumers had behaviors that were considered unsafe, the predicted outcome for both was a sole case of mild salmonellosis per 800 meals consumed in this manner. Thus, if they both ate 50 such egg meals per year, this would equate to one case of mild salmonellosis in 16 years.

When assessing risk of salmonellosis, it might be best to focus on cases of hospitalization and death [11,16]. First, because like vaccination against *Salmonella* [47,48], mild illness from *Salmonella* increases immunity and reduces severity of future cases of salmonellosis [49]. Second, most (97%) of the cost of salmonellosis is associated with hospitalization and death and not mild illness (3%) [50]. Third, it is easier to track hospitalizations and deaths from *Salmonella* than illnesses, which are under reported [51]. Consequently, it makes sense to focus on reducing hospitalizations and deaths from salmonellosis and to consider mild illnesses as events that reduce cases of severe salmonellosis (hospitalization, death) via greater resistance to salmonellosis. The role of secondary transmission of *Salmonella* from fecal shedding in cases of infection and illness on risk and severity of salmonellosis from chicken eggs was not simulated in the current study because of data and knowledge gaps. Thus, the impact on the risk predictions is unknown.

## 4. Conclusions

The data collection and modeling methods of the Poultry Food Assess Risk Model (PFARM) for *Salmonella* were adapted to local resources of the Saudi Food and Drug Authority and used to assess the risk and severity of salmonellosis for a cohort of 125 chicken egg consumers in Riyadh, Saudi Arabia. This was the first PFARM study to use real-world data from the PFARM consumer survey to assess the risk and severity of salmonellosis from poultry food. Also, the PFARM, WSE-qPCR method for *Salmonella* enumeration in poultry meat was successful in a new food matrix (chicken eggs) using a different commercial qPCR test kit for *Salmonella*. In addition, the PFARM was successfully modified to include a new step (microbial growth during home storage) in the exposure pathway for assessing the dose of *Salmonella* consumed.

The next steps are to improve the PFARM consumer survey and chicken egg testing data for *Salmonella* and then apply the PFARM data collection and modeling methods more broadly to chicken egg production chains and consumers in Saudi Arabia for the purpose of better defining the problem and its control. Key to this is the ability of the PFARM to find combinations of risk factors that cause salmonellosis and that can be targeted for mitigation of this risk to human health.

Although unsafe behaviors (temperature abuse, poor hygiene, and undercooking of eggs) were observed in the consumer cohort investigated and simulated in the PFARM, only two cases of mild salmonellosis and no cases of severe salmonellosis (hospitalization, death) per 100,000 egg meals were predicted by the PFARM in this study. Thus, the chicken eggs investigated were safe to eat despite significant *Salmonella* contamination at retail and significant high risk food safety behaviors by the consumer cohort during home storage and preparation of the egg meals.

## Figures and Tables

**Table 1:** Results of the whole sample enrichment, quantitative polymerase chain reaction (WSE-qPCR) assay for enumeration and cultural isolation and serotyping of *Salmonella* in shell eggs in Riyadh, Saudi Arabia.

		*Salmonella*			
Date		log_10_ Growth Units/Egg			
(dd.mm.yyyy)	C_T_ ^a^	Lower	X	Upper	Serotype
08.05.2023	25	2.009	2.299	2.571	Cerro
15.05.2023	ND		0 ^b^		Stanley
15.05.2023	35	0.000	0.061	0.369	Cerro
22.05.2023	ND		0 ^b^		Cerro
22.05.2023	33	0.000	0.431	0.723	Cerro
22.05.2023	26	1.753	2.047	2.325	Enteritidis
22.05.2023	22	2.808	3.077	3.338	Winston

^a^ Abbreviations: C_T_ = cycle threshold value; ND = not detected. ^b^ Assumed level in non-detected samples.

**Table 2:** Summary of consumer survey results for the Poultry Food Assess Risk Model (PFARM) for *Salmonella* and chicken eggs in Riyadh, Saudi Arabia, from very low (1) to very high (5) risk.

Consumer Survey		Risk Category					
Code	Item	1	2	3	4	5	Mean
S1	Home storage time ^a^	2	16	33	38	36	3.7
S2	Home storage temperature ^b^	88	32	5	0	0	1.3
S3	Hygiene	42	40	32	9	2	2.1
S4	Meal preparation time ^c^	115	10	0	0	0	1.1
S5	Kitchen temperature ^d^	45	47	29	3	1	1.9
S6	Fried egg doneness ^e^	49	35	20	20	1	2.1
S7	Portion size ^f^	28	47	32	10	8	2.4
S8	Food consumption behavior	53	49	16	4	3	1.8
S9	Consumer health and immunity	0	38	44	33	10	3.1

^a^ 1 = 0 to 1; 2 = 1 to 4; 3 = 4 to 7; 4 = 7 to 14; 5 = 14 to 21 days. ^b^ 1 = < 8; 2 = 8 to 20; 3 =20 to 30; 4 = 30 to 35; 5 = 35 to 40 °C. ^c^ 1 = < 1; 2 = 1 to 2; 3 =2 to 4; 4 = 4 to 6; 5 = 6 to 8 h. ^d^ 1 = 16 to 22; 2 = 22 to 25; 3 = 25 to 30; 4 = 30 to 35; 5 = 35 to 40 °C. ^e^ 1 = over hard; 2 = over medium; 3 = over easy; 4 = sunny side up; 5 = raw. ^f^ 1 = 1 egg; 2 = 2 eggs; 3 = 3 eggs; 4 = 4 eggs; 5 = 5 eggs.

**Table 3:** Consumer response from replicate simulations of the Poultry Food Assess Risk model for *Salmonella* and chicken eggs in Saudi Arabia.

Seed	No Exposure	No Response	Infection	Illness	Hospital	Death
1	12,337	147	15	1	0	0
8	12,358	136	6	0	0	0
11	12,355	136	9	0	0	0
28	12,327	158	14	1	0	0
32	12,354	133	13	0	0	0
46	12,320	163	17	0	0	0
52	12,334	159	7	0	0	0
69	12,345	148	7	0	0	0
total	98,730	1180	88	2	0	0

**Table 4:** Model outputs for egg meals resulting in cases of salmonellosis as predicted by the Poultry Food Assess Risk Model for *Salmonella* and chicken eggs in Saudi Arabia.

			Consumer 59	Consumer 22
Yolk Membrane Integrity Lost	Retail	fraction	0.61	0.40
*Salmonella*	Retail	per egg meal	132	257
*Salmonella*	Home Storage	per egg meal	1,301,715	179,073,773
*Salmonella*	Kitchen Fomites	per egg meal	650	931,183
*Salmonella*	Lettuce	per egg meal	0	100,940
*Salmonella*	Abused Lettuce	per egg meal	0	100,940
*Salmonella*	Egg(s)	per egg meal	6896	0
Native Microflora	Retail	per egg meal	77,253	234,699
Native Microflora	After Home Storage	per egg meal	18,600,000,000	18,600,000,000
Native Microflora	Kitchen Fomites	per egg meal	2,388,527	96,226,770
Native Microflora	Lettuce	per egg meal	1738	9,605,058
Native Microflora	Consumption	per egg meal	89,055,082	279,895
Consumer Survey	Home Storage Time	Risk Category	4	4
Consumer Survey	Home Storage Temperature	Risk Category	2	3
Consumer Survey	Hygiene	Risk Category	1	4
Consumer Survey	Meal Preparation Time	Risk Category	2	1
Consumer Survey	Kitchen Temperature	Risk Category	1	3
Consumer Survey	Egg Doneness	Risk Category	4	3
Consumer Survey	Portion Size	Risk Category	3	3
Consumer Survey	Food Consumption Behavior	Risk Category	3	3
Consumer Survey	Consumer Health and Immunity	Risk Category	3	2
Consumption	Food Consumption Behavior	Score	1.8	1.8
Consumption	Consumer Health and Immunity	Score	2.6	1.2
Consumption	Zoonotic Potential	Score	5.0	5.0
Consumption	Disease Triangle Score	Score	9.4	7.9
Consumption	Dose Consumed	per egg meal	6896	100,940
Consumption	Illness Dose	per egg meal	6109	84,883
Consumption	Consumer response	illness	1.13	1.19
Consumption	Consumer Response	per egg meal	1.13	1.19

## Data Availability

The original contributions presented in the study are included in the article/Supplementary Materials. Further inquiries can be directed to the corresponding authors.

## Supplementary Materials

The following supporting information can be downloaded at https://www.mdpi.com/article/10.3390/foods14193382/s1; The Poultry Food Assess Risk Model for Salmonella and chicken eggs.
